# Plasma Prostaglandin E_2_ Levels Correlated with the Prevention of Intravenous Immunoglobulin Resistance and Coronary Artery Lesions Formation via CD40L in Kawasaki Disease

**DOI:** 10.1371/journal.pone.0161265

**Published:** 2016-08-15

**Authors:** Ho-Chang Kuo, Chih-Lu Wang, Kuender D. Yang, Mao-Hung Lo, Kai-Sheng Hsieh, Sung-Chou Li, Ying-Hsien Huang

**Affiliations:** 1 Department of Pediatrics, Kaohsiung Chang Gung Memorial Hospital and Chang Gung University College of Medicine, Kaohsiung, Taiwan; 2 Kawasaki Disease Center, Kaohsiung Chang Gung Memorial Hospital, Kaohsiung, Taiwan; 3 Department of Pediatrics, Po-Jen Hospital, Kaohsiung, Taiwan; 4 Institute of Biomedical Sciences, Mackay Medical School and Department of Pediatrics, Mackay Memorial Hospital, Taipei, Taiwan; 5 Genomics and Proteomics Core Laboratory, Department of Medical Research, Kaohsiung Chang Gung Memorial Hospital and Chang Gung University College of Medicine, Kaohsiung, Taiwan; Institut National de la Santeet de la Recherche Medicale (INSERM), FRANCE

## Abstract

**Background:**

A form of systemic vasculitis, Kawasaki disease (KD) occurs most frequently in children under the age of five years old. Previous studies have found that Prostaglandin E_2_ (PGE_2_) correlates with KD, although the related mechanisms are still unknown. CD40L may also be a marker of vasculitis in KD, so this study focuses on PGE_2_ and CD40L expression in KD.

**Materials and Methods:**

This study consisted of a total of 144 KD patients, whose intravenous immunoglobulin (IVIG)/coronary arterial lesion (CAL) formation resistance was evaluated. PGE_2_ levels were evaluated *in vitro* to study the effect of CD40L on CD4+ T lymphocytes.

**Results:**

PGE_2_ levels significantly increased after IVIG treatment (p<0.05), especially in patients who responded to initial IVIG treatment (p = 0.004) and for patients without CAL formation (p = 0.016). Furthermore, an *in vitro* study revealed that IVIG acted as a trigger for PGE_2_ expression in the acute-stage mononuclear cells of KD patients. According to our findings, both IVIG and PGE_2_ can impede surface CD40L expressions on CD4+ T lymphocytes (p<0.05).

**Conclusions:**

The results of this study are among the first to find that plasma PGE_2_ is correlated with the prevention of IVIG resistance and CAL formation through CD40L in KD.

## Introduction

Kawasaki disease (KD) is a form of systemic vasculitis that was initially described by Tomisaki Kawasaki in 1974[1] and later reported in English. KD occurs throughout the world and generally affects children under the age of five years old. Its most serious complication is coronary artery lesions (CAL)[2], which includes coronary artery fistulas and coronary artery aneurysms [3], and is the primary reason that children develop heart disease [3–5]. Treatment typically involves intravenous immunoglobulin (IVIG) therapy (2 g/kg/dose) combined with high-dose aspirin (80~100 mg/kg/day), which has successfully reduced the prevalence of coronary artery aneurysms in KD patients from 20% to 3–5% [6–8].

Although the cause of KD is not yet known, recent studies have found that endothelial dysfunction (ED) may be a driving force in the progression of KD [9, 10]. PGE_2_ can both expand the coronary arteries and enhance vascular permeability through four receptor subtypes (EP1, EP2, EP3, and EP4) in a complex way [11], suppress T cell receptor signals, and help resolve inflammation [12]. Some previous studies have already investigated the function of PGE_2_ in relation to KD [13–15]. Lee et al. (1988) was the first to show that PGE_2_ plasma levels increased considerably in acute-stage KD and then decreased during the recovery stage in 15 KD patients within their study [13]. Furthermore, another study found that PGE_2_ could activate β1-integrins through the PGE_2_ receptor in human coronary arterial endothelial cells [14]. This study also observed that PGE_2_ often functions via the EP2 receptor in HCAEC and showed that the EP2 antagonist may be able to control the inflammatory response of KD [14]. Meanwhile, prostacyclin analogue has been successfully used to save the extremities of a KD patient with peripheral gangrene [16]. Furthermore, single nucleotide polymorphisms of an ATP-binding cassette, subfamily C, member 4, which is a mediator of prostaglandin efflux, are correlated with KD susceptibility [17]. These findings piqued our interest in the influence of PGE_2_ on the pathogenesis of KD, and thus this study aims to determine the specific role of PGE_2_ in both KD’s pathophysiology and its treatment outcomes.

CD40 Ligand (CD40L) is part of the TNF family and is vital to the vascular system’s pathophysiology [18]. In the course of this research, we found both an elevated expression of CD40 ligand (CD40L) on CD4+ helper T cells and platelets in acute-stage KD, as well as a considerably higher expression in KD patients with CAL [19]. Although PGE_2_ has been proven to inhibit CD40L expression on activated neonatal T cells [20], the clinical importance of both PGE2 and CD40L in KD patients has yet to be properly defined. Furthermore, CD40 and CD40L gene polymorphisms confirmed the association between susceptibility and CAL of KD [21–23]. Researching the plasma PGE_2_ levels at three different stages of KD and carrying out an *in vitro* study of primary mononuclear cells from acute-stage KD patients have allowed us to determine the precise role of PEG_2_ and its relationship with CD40L with regard to the disease outcome of KD patients.

## Materials and Method

### Patients

A total of 144 KD patients from Kaohsiung Chang Gung Memorial Children’s Hospital in Taiwan from 2007 to 2009 participated in this study. They were all children that met the KD criteria [24, 25] and who were treated with IVIG at the hospital. We also found 50 age-matched febrile control patients that had been admitted to the hospital with a respiratory tract infection, including acute pharyngitis, acute tonsillitis, croup, acute bronchitis, and acute bronchiolitis. Peripheral blood samples were taken at three times: before IVIG treatment (pre-IVIG) and within three days after completing initial IVIG treatment (post-IVIG< 3 days), which served as the acute stage samples, as well as at least three weeks after IVIG treatment, which functioned as the subacute stage samples (post-IVIG> 3 weeks), as described earlier in this paper [26]. CAL formation is defined as a coronary artery with an internal diameter that measured at least 3 mm (4 mm if the patient was older than five years old) or an internal diameter of a segment that was at least 1.5 times that of an adjacent segment, as observed in an echocardiogram [7, 27]. IVIG responsiveness is defined as fever reduction within 48 h of completing IVIG treatment without relapse (temperature >38°C) for at least seven days, as well as obvious improvement or normalization of inflammation [3, 7, 28]. This study was approved by the Chang Gung Memorial Hospital’s Institutional Review Board, and we obtained the written informed consent from the parents or guardians of all the subjects. All of the methods used complied with the approved relevant guidelines.

### Plasma PGE_2_ measurements by enzyme-linked immunoassay (ELISA)

We used the ELISA kit (R&D Systems, Minneapolis, MN) in accordance with the manufacturer's instructions in order to determine the plasma PGE_2_ levels.

### Human mononuclear cell isolation

We freshly isolated peripheral blood mononuclear cells (PBMC) from whole blood using the previously described Ficoll-Paque separation method (Pharmacia, Uppsala, Sweden) [29]. For monocytes isolation, said cells were put in a 100-mm dish (Becton Dickinson, Franklin Lakes, NJ) and allowed to adhere in a 5% CO2 incubator at 37°C for 2 hours. We removed the cells that did not adhere, while the adherent cells were carefully washed at least twice with warm PBS (Biochrom AG) before being harvested. The purity of the resulting cell suspension was then randomly tested using fluorescence-activated cell sorter (FACS) analysis; the sample was deemed pure if it yielded at least 95% monocytes as previous described [30]. Afterward, various concentrations (5, 25, 250, and 2500 mg/dL) of IVIG (7S-IVIG; Gamimmune-N, Bayer, USA) or recombinant PGE_2_ (0, 10^−6^, 10^−5^, and 10^-4^M, R&D) were incorporated into the PBMC (with monocyte isolation) and PBMC (without monocyte isoloation) for assay of PGE2 and CD40L expression on CD4+ T cells, in a time series of 0.5, 1, 6, 24, and 48 hours, respectively. All tests were separately carried out three times each.

### Detecting CD40L expression on CD4+T-Cells

Peripheral venous blood samples were drawn into sterile tubes containing heparin (Becton Dickinson, Heidelberg, Germany). Within 1 hour, 200 μL of whole blood was mixed with 20 μL of appropriate monoclonal antibody conjugates for 30 minutes (4°C in darkness). Anti-CD4 fluorescein isothiocyanate (FITC) (Becton Dickinson) and anti-CD40L phycoerythrin (PE) (Ancell Group, Bayport, MN) were used for staining, while isotype-matched FITC- and PE-conjugated mouse IgG1 (Pharmigen, San Diego, CA) were used as the negative controls. We chose a protein kinase C activator phorbol myristate acetate (PMA, 32 nM) and calcium ionophore (A23187; 1μg/mL) to promote CD40L expression on CD4+ T cells. Following 4 hours of incubation, each sample was collected with a red blood cell lysing buffer (Becton Dickinson), washed two times with cold phosphate-buffered saline (PBS), fixed with 1% paraformaldehyde, and inspected with a FAScan [19]. A total of 10^4^ cells were acquired and inspected using CellQuest software (Becton Dickinson), resulting in cell viability above 95%.

### Statistical analysis

All data are presented as mean ± standard error. We analyzed quantitative data with Student’s t-test or, when appropriate, one-way ANOVA; we evaluated any data changes before and after IVIG treatment using the paired sample *t-*test. A two-sided p-value less than 0.05 is considered statistically significant. All statistical analyses were performed using SPSS version 22.0 for Windows XP (SPSS, Inc., Chicago, USA).

## Results

### Patient characteristics

Of the 144 KD patients enrolled in this study, 93 (64.6%) were male. The mean age was 1.8±0.15 years old. As for treatment, 130 patients (90.2%) received one dose of IVIG therapy, while the other 14 patients (9.8%) received two IVIG treatments. The echocardiograms revealed that 44 patients (30%) had CAL throughout the entire course of the disease, 11 of which (25%) had received two doses of IVIG therapy (78.6%).

### Plasma PGE2 levels in KD patients

**Fig 1:**
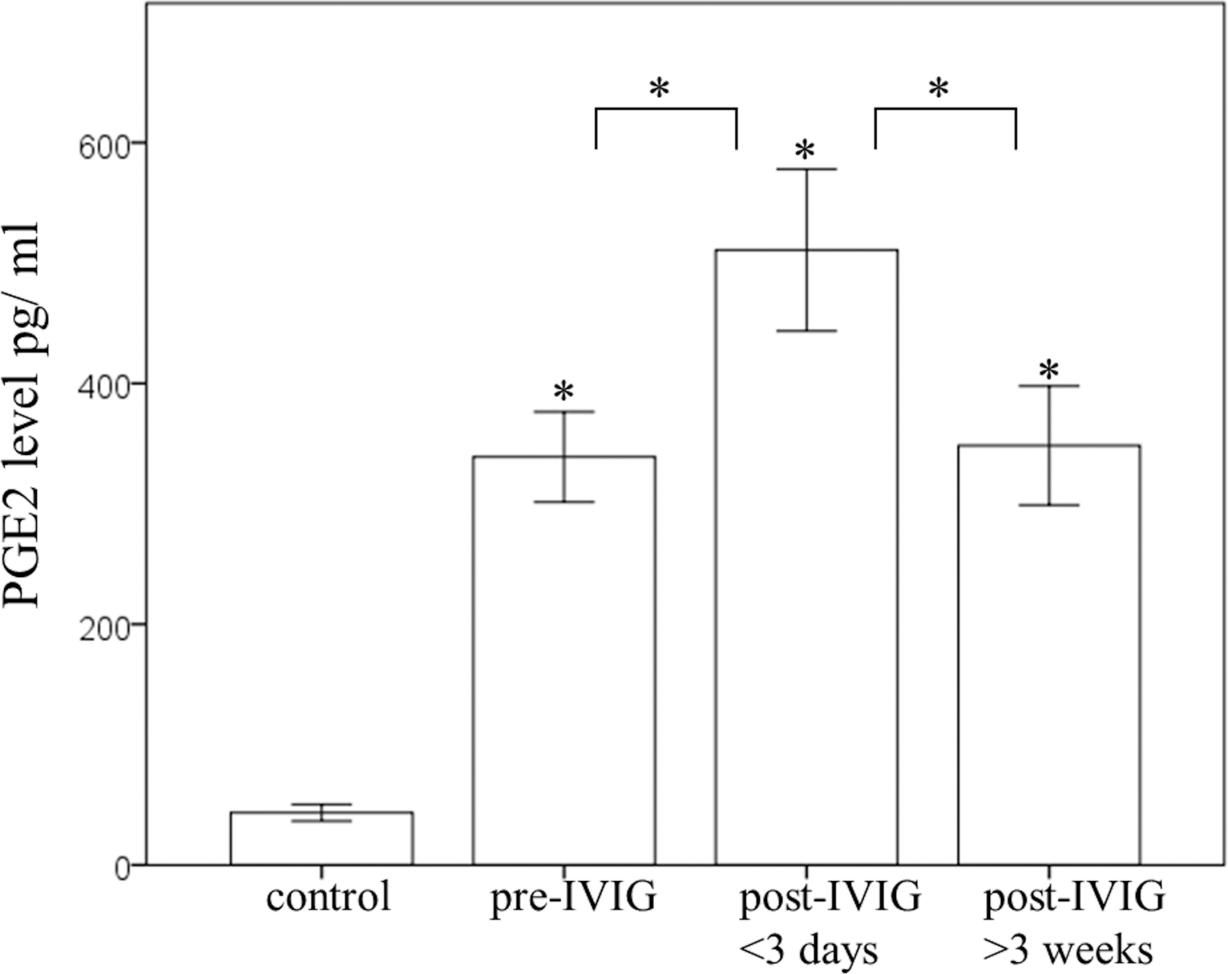
A comparison of plasma PGE_2_ levels using ELISA for 15 controls and different stages of Kawasaki disease (KD) (N = 144). PGE_2_ levels were higher in all three stages of KD patients than in the control subjects. PGE_2_ levels significantly increased after patients underwent intravenous immunoglobulin (IVIG) treatment and then decreased during the subacute stage, which was defined as at least three weeks after KD patients completed the IVIG treatment. *indicates p < 0.05 between the control and KD groups. Data are presented as mean ± standard error.

We used ELISA to determine the plasma PGE_2_ protein levels of the study’s participants. Fig 1 reveals that PGE_2_ levels were more elevated during all three stages of KD patients than in the control subjects (all p< 0.001). Furthermore, the PGE_2_ levels significantly increased following IVIG treatment (355 ± 32 pg/ml and 511 ± 72 pg/ml, p = 0.004) and then decreased again during the subacute stage (376 ± 40 pg/ml), which was defined as at least three weeks after KD patients completed treatment with IVIG. We demonstrated that the PGE_2_ plasma levels did not significantly differ before IVIG treatment and during the subacute stage (p = 0.502).

### Differences in plasma PGE_2_ levels related to IVIG treatment response and CAL in KD patients

**Fig 2:**
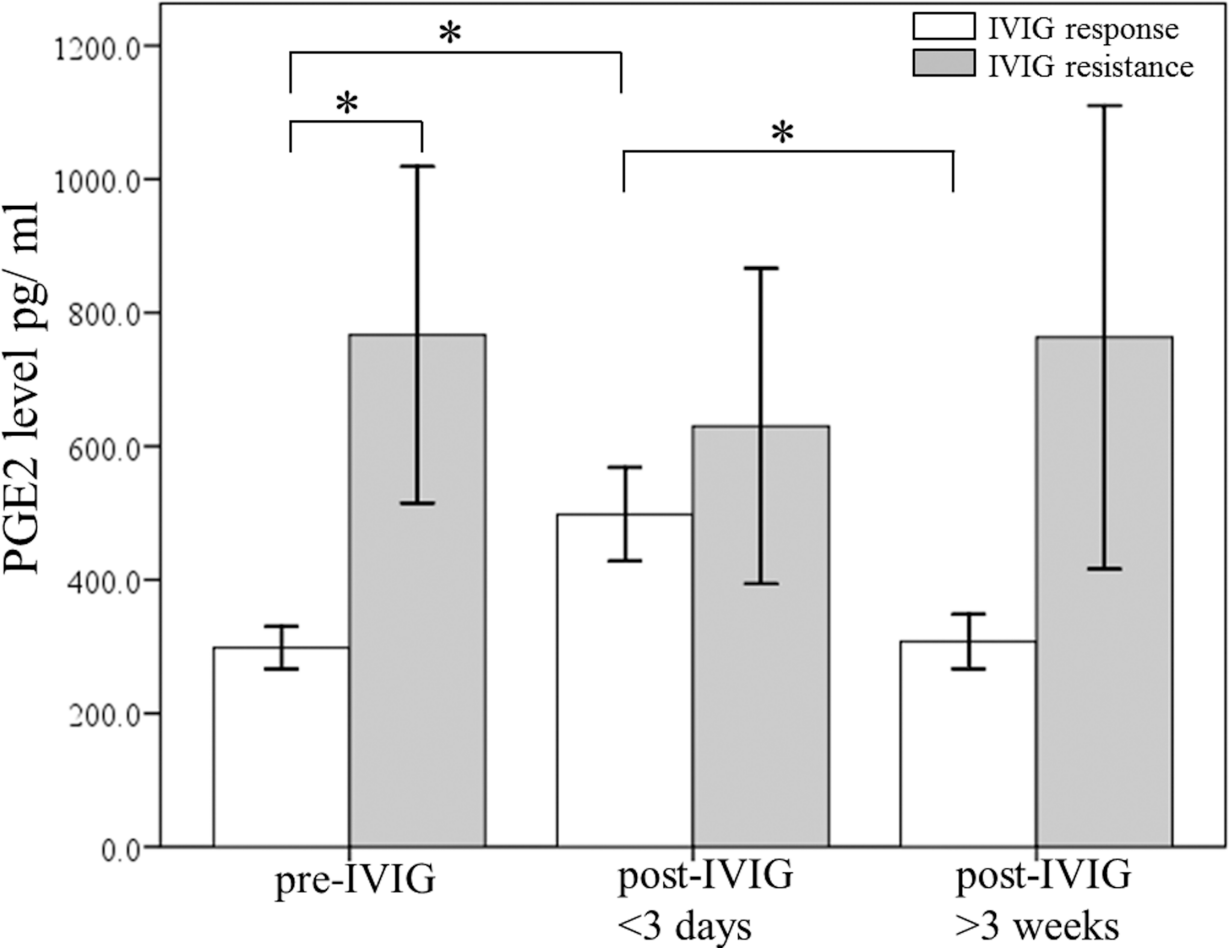
A comparison of plasma PGE_2_ levels using ELISA between KD patients that responded to IVIG treatment (N = 130) and those that were IVIG resistant (N = 14) prior to receiving IVIG treatment and following IVIG treatment. *p < 0.05. Data are presented as mean ± standard error.

**Fig 3:**
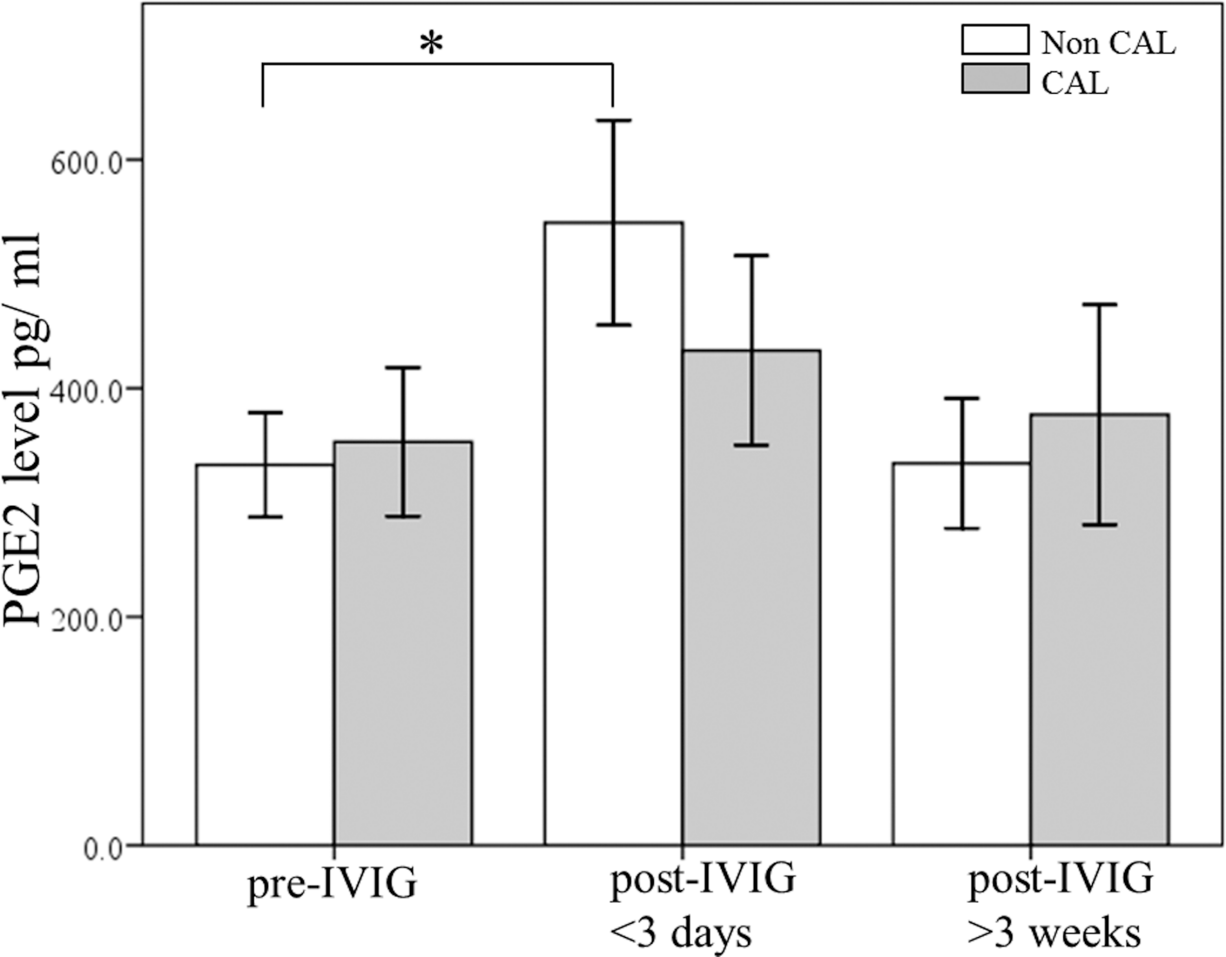
A comparison of plasma PGE_2_ levels using ELISA between patients with KD without (n = 100) and with (n = 44) coronary arterial lesions (CAL) prior to receiving IVIG treatment and after IVIG treatment. *p < 0.05. Data are presented as mean ± standard error.

Widespread inflammation is frequently correlated with the occurrence of KD and the subsequent development of CAL, and PGE_2_ is a key indicator of the anti-inflammatory effect [31, 32]. In order to determine the changes in PGE_2_ levels after IVIG treatment on CAL formation and its following treatment response, the participating KD patients were divided into two categories: the IVIG resistant group and the IVIG responsive group. Furthermore, Fig 2 shows that the PGE_2_ levels were higher in the IVIG resistant group than in the IVIG responsive group prior to undergoing IVIG treatment (p = 0.032). We also observed a significant increase in the PGE_2_ levels of the IVIG responsive group (p = 0.004), but no significant increase of PGE_2_ levels was found in the IVIG resistance group (p = 0.776). The PGE2 levels of the IVIG-resistant patients were extraordinarily higher during all three time periods and did not differ throughout IVIG treatment. Finally, Fig 3 demonstrates that the PGE_2_ plasma levels significantly increased following IVIG treatment among the non-CAL group (p = 0.016), while we found no statistical PGE_2_ increase following IVIG among the CAL group (p = 0.08).

### IVIG regulation of PGE_2_ expression in PBMC

**Fig 4:**
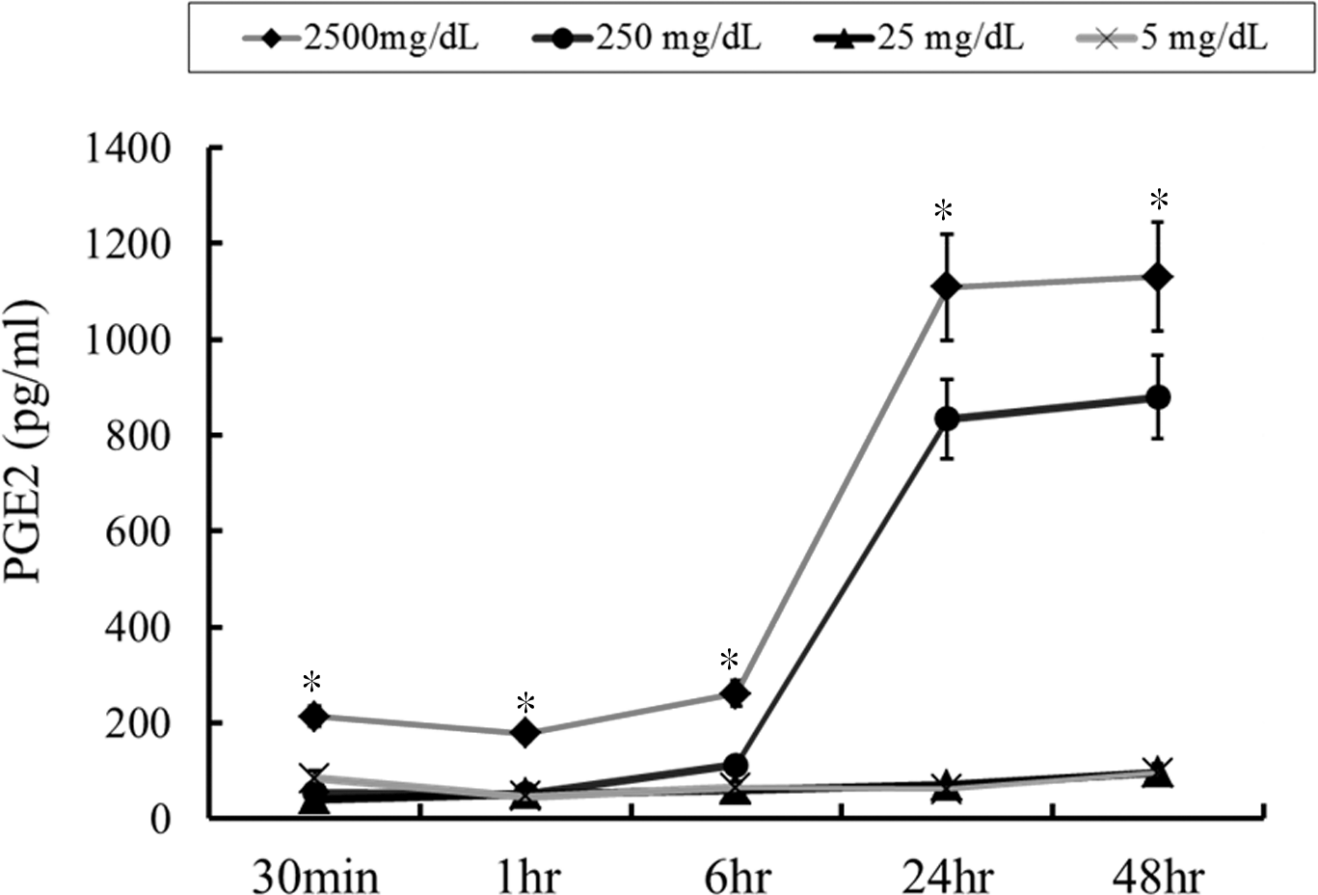
IVIG regulation of PGE_2_ expression in peripheral blood mononuclear cells. IVIG significantly triggers PGE_2_ expression in a time-dependent and dose-dependent manner in peripheral blood mononuclear cells. *p < 0.05 compared to all other groups. Data are presented as mean ± standard error. All tests were separately carried out three times each (N = 4).

Then PBMC was treated in acute-stage KD patients (N = 4) with various IVIG concentrations in order to evaluate whether IVIG influences PGE_2_ expression. As predicted, IVIG triggered PGE_2_ expression considerably in a time-dependent and concentration-dependent manner in PBMC (Fig 4). High dose IVIG (2500mg/dL and 250mg/dL) in 24 and 48 hours significant stimulated PGE2 expression but not found in low dose IVIG (25mg/dL and 5mg/dL). In order to evaluate the importance of monocyte among PBMC in the mechanism of IVIG stimulation PGE2 expression, we removed monocye from PMBC for further study. After removed monocyte from PBMC, we cannot found the significant increase of PGE2 after high dose IVIG stimulation (N = 4, data not shown).

### IVIG inhibits CD40L expression on CD4^+^ T cells

**Fig 5:**
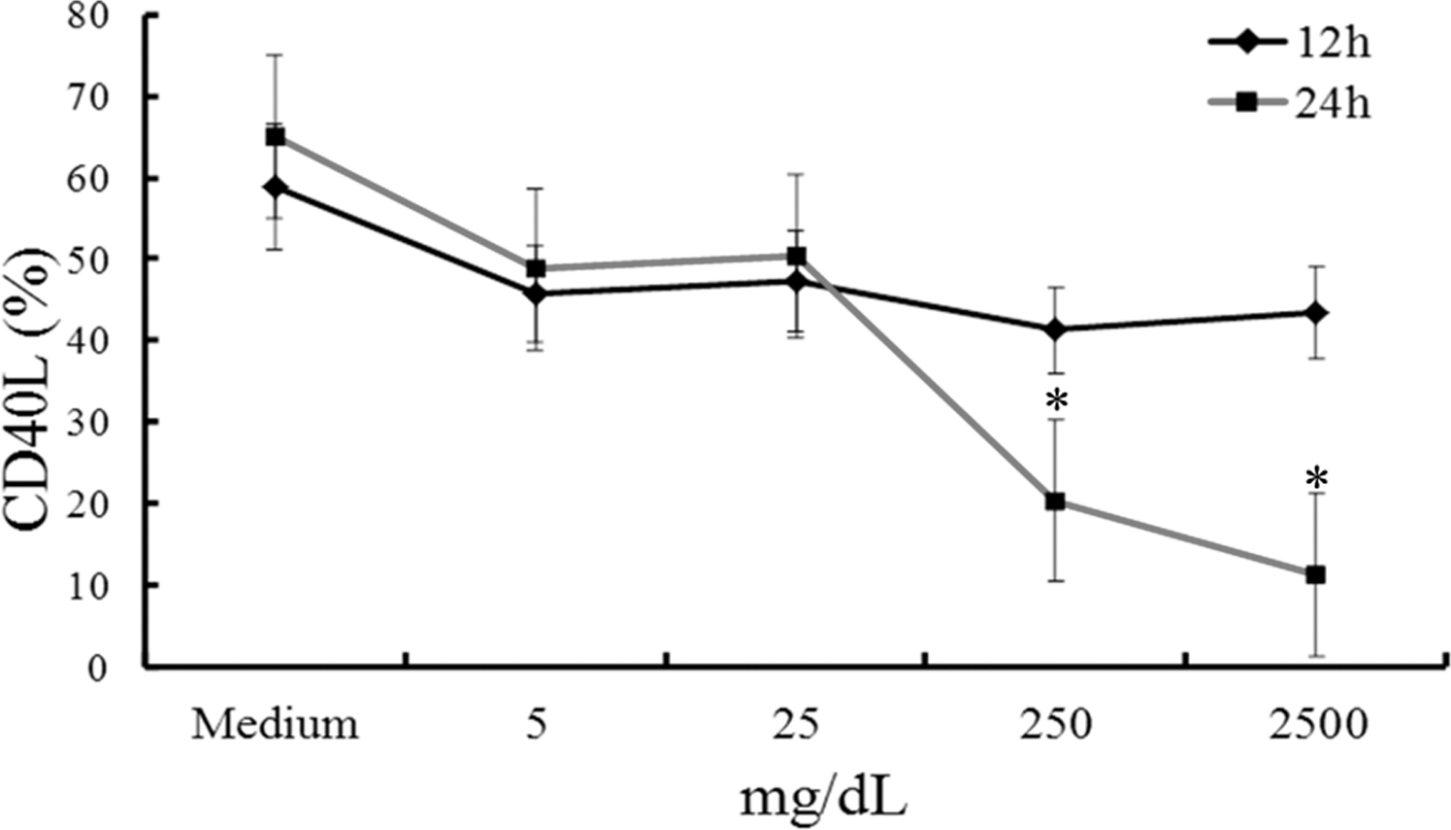
IVIG inhibition of CD40L expression on CD4+ T cells. Greater concentrations (250 mg/dL and 2500 mg/dL) of IVIG significantly inhibited CD40L expression on CD4+ T cells after 24 hours of treatment.*p < 0.05 compared to the average. Data are presented as mean ± standard error. All tests were separately carried out three times each (N = 4).

Our research team has already found that an elevated CD40L expression on CD4+ T cells and platelets in acute-stage KD combined with IVIG therapy can successfully reduce CD40L expression in KD patients [19]. Furthermore, we have explored whether IVIG had an affected CD40L expression on CD4+ T cells *in vitro*. As Fig 5 shows, after an *in vitro* stimulation by PMA and A23187 for 4 hours, a greater concentration of IVIG was able to significantly inhibit CD40L expression on CD4+ T cells with 24 hours of treatment.

### Recombinant PGE_2_ inhibits CD40L expression on CD4^+^ T-Cells

**Fig 6:**
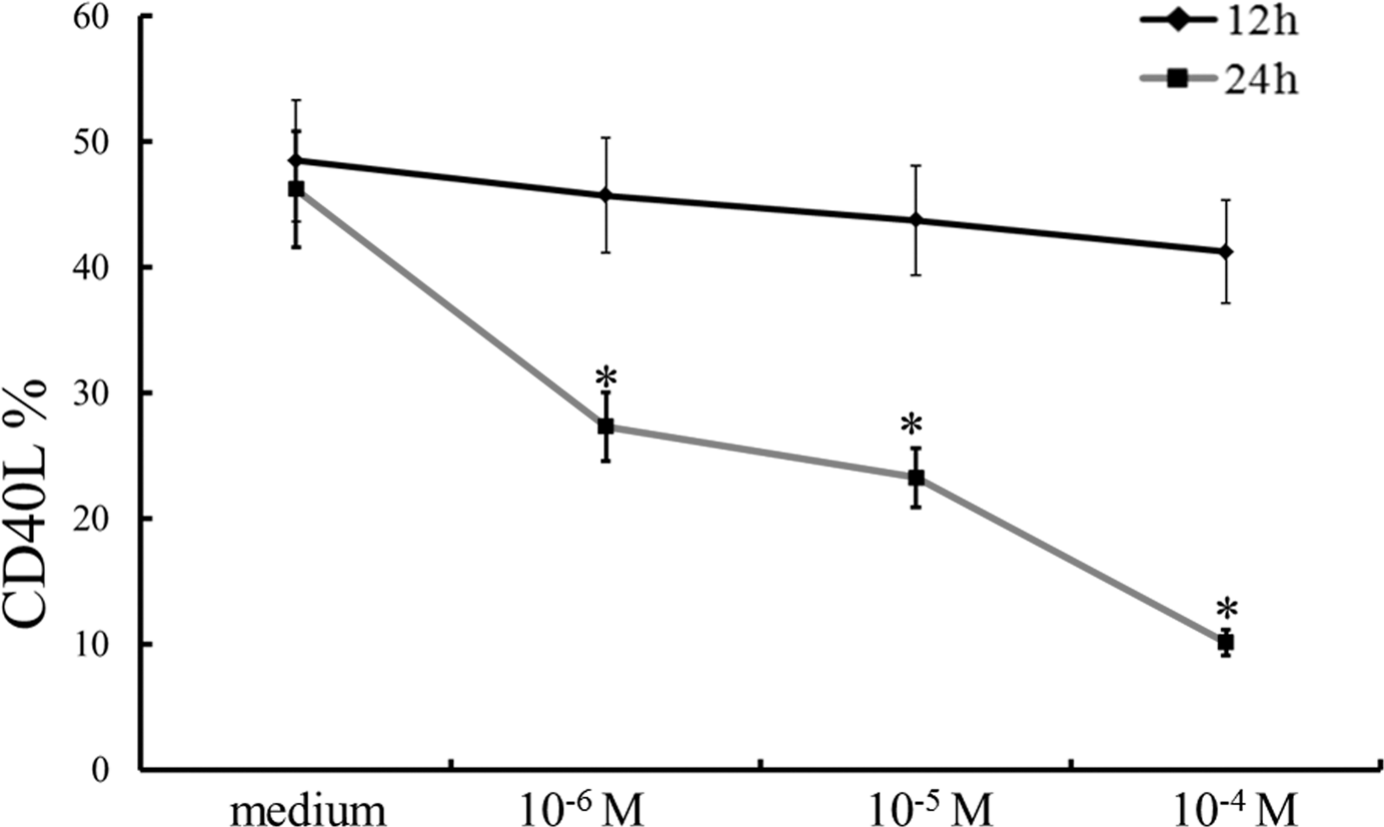
Recombinant PGE_2_ prevention of CD40L expression on CD4+ T cells. PGE_2_ significantly hinders CD40L expression on CD4**+**T cells in a dose-dependent manner after 24 hours of treatment. *p < 0.05 compared to the average. Data are presented as mean ± standard error. All tests were separately carried out three times each (N = 4).

This study demonstrated that plasma PGE_2_ levels did not elevate after IVIG treatment, which correlated with both IVIG resistance and CAL in KD patients. Furthermore, CD40L expression on CD4**+** T cells and platelets was related to the manifestation of CAL in KD patients [19]. To determine whether PGE_2_ can control CD40L expression on CD4**+** T cells, various concentrations of recombinant PGE_2_ were integrated into PBMC after *in vitro* stimulation by PMA and A23187. Fig 6 demonstrates that PGE_2_ can inhibit CD40L expression on CD4**+** T cells with a dose-dependent method with 24 hours of treatment.

## Discussion

To the best of our knowledge, this is the first study to report that an increase of PGE_2_ levels following IVIG treatment was observed in patients that responded to IVIG (p = 0.004) and in those without CAL formation (p = 0.016). On the other hand, a significant post-IVIG increase in plasma PGE2 levels was not found in either IVIG non-responders or in patients who had manifested CAL formation. An additional *in vitro* study confirmed that IVIG could trigger PGE_2_ expression in PBMC, as well as that both IVIG and PGE2 were capable of blocking CD40L expression on CD4**+** T cells in a dose-dependent and time-dependent way.

PGE_2_ has powerful immunosuppressive characteristics, one of which is blocking the creation of oxygen radical species, leukocyte chemotaxis, proinflammatory cytokines, and chemokines [32, 33]. As a form of vasculitis, KD frequently consists of elevated inflammatory markers and IVIG resistance, which may be related to the development of CAL in KD patients [34–36]. IVIG is well known to exert anti-inflammatory properties. The current study found that PGE_2_ levels were significantly higher in the participating KD patients upon completing IVIG therapy, as seen in an *in vitro* study of IVIG stimulation in PBMC from KD patients. Our research team recently found that KD was also related to higher IL-17A and IL-6 levels and that IVIG resistance was related to higher levels of IL-17A [37]. Our findings agree with those of *Saha et al*., who found that PGE_2_ negatively normalizes the production of inflammatory cytokines/chemokines and IL-17 in visceral leishmaniasis [38]. Furthermore, PGE_2_ can enhance Th17 and Th1 cell development [39] and KD was shown to be related to the imbalance of T helper (Th) 17 cells and Treg cells, as well as the down-regulation of the Treg transcription factor FoxP3 expression in KD patients [37, 40]. However, IVIG could promote PGE_2_ to favor Treg expansion, but can also inhibit Th17 and Th1 cell development [41–44]. In a recent study, our group also demonstrated that IVIG was capable of increasing the expression of Treg-transcription factor FoxP3 in KD patients [37]. In agreement with our findings, the inhibitory activity on toll like receptor agonist-induced IFNα production [45] and the expansion of Treg cells in plasmacytoid dendritic cells or autoimmune patients by IVIG are also required with induction PGE_2_ levels [46, 47]. A significant increase was also seen in the IP-10 levels of KD patients, which normalized following IVIG treatment [35]. IP-10 was also shown to considerably trigger NK cell cytotoxicity in cases in which immunosuppression by PGE_2_ took place [48]. In our study, we found that change of PGE_2_ was a more important predictor of KD outcomes than the actual levels prior to IVIG treatment, as well as an original observation that the lack of increase in PGE_2_ levels following IVIG treatment correlates with CAL formation and IVIG resistance. By this IVIG brings about immune balance and PGE_2_ is crucial to immunosuppression in KD patients.

High-dose aspirin is currently the standard treatment for acute-phase KD patients [49]. Inactivation of the cyclooxygenase can hinder the production of prostaglandins. The plasma prostaglandin levels in the high-dose aspirin group were shown to be lower than that in the low-dose aspirin group of KD [50]. Likewise, we discovered that treating KD patients with high-dose aspirin can impair the improvement of the inflammatory markers after IVIG therapy, but the disease outcomes are unaffected [25]. Therefore, high-dose aspirin does not offer any appreciable benefits to acute-phase KD.

The relationship between CD40L and its CD40 receptor is key to controlling inflammatory and immune responses by activating tissue structure cells, such as endothelial cells, smooth muscle epithelial cells, and fibroblasts [51, 52]. We also observed that the CD40L expression on CD4**+** T cells was significantly higher in KD patients than in the febrile controls and then significantly decreased three days after completing IVIG therapy [19]. More importantly, CD40L expression on CD4**+** T cells and platelets correlated significantly with the manifestation of CAL but CD40L expression on CD8**+** T cells or soluble CD40L did not, indicating that CD40L on CD4**+** T cells and platelets may be vital to the immunopathogenesis of CAL through interaction with CD40-positive target cells and activation of the immune system and elicit inflammatory reactions, thus resulting in vascular endothelial damage. An *in vitro* study found that both IVIG and PGE_2_ could prevent surface CD40L expression on CD4+ T lymphocytes, results that agree with the previous finding that the difference in CD40L expression on CD4+ T lymphocytes after IVIG correlated with CAL in KD. PGE_2_ and CD40-CD40L signaling have also been shown to have potential effects in patients with atherosclerotic vascular diseases, which helps to explain the increased potential risk for atherosclerosis in KD patients [52–54].

This study’s results are among the first to provide a mechanism to explain the relationships of IVIG/PGE_2_/CD40L among KD patients. Better comprehension of the fundamental mechanisms of the relationships between IVIG/PGE_2_/CD40L pathways and long-term coronary arterial remodeling can ultimately result in more effective and innovative treatments for KD patients.

### Conclusion

Our findings are foremost in providing proof that a change of plasma PGE_2_ levels following IVIG treatment was related to IVIG resistance and CAL in KD patients by managing surface CD40L on CD4+ T lymphocytes.

## Abbreviations

CAL: coronary artery lesions
ELISA: enzyme-linked immunoassay
IVIG: intravenous immunoglobulin
KD: Kawasaki disease
PGE_2_: Prostaglandin E_2_
